# ECPR for cardiac arrest caused by abnormal uterine bleeding and coronary vasospasm: a case report

**DOI:** 10.3389/fcvm.2024.1481498

**Published:** 2024-12-20

**Authors:** Qiping Sheng, Yingjie Wang, Zhiyang Wu, Xiangyang Zhao, Dawei Wu, Zhi Li, Xi Guo

**Affiliations:** Department of Critical Care Medicine, Cheeloo College of Medicine, Qilu Hospital (Qingdao), Shandong University, Qingdao, Shandong, China

**Keywords:** uterine bleeding, extracorporeal cardiopulmonary resuscitation, coronary vasospasm, cardiac arrest, extracorporeal membrane oxygenation

## Abstract

**Introduction:**

Cardiac arrest during pregnancy is receiving increasing attention. However, there are few reports on cardiac arrest in nonpregnant women caused by abnormal uterine bleeding (AUB). We report a case in which extracorporeal cardiopulmonary resuscitation (ECPR) was used in a patient with cardiac arrest caused by AUB and coronary vasospasm.

**Patient presentation:**

A 52-year-old female patient presented to the emergency department because of sudden chest pain, with a history of hypertension, coronary heart disease and AUB for more than half a month. At the initial stage of admission, cardiac arrest occurred after the ECG demonstrated ST-segment elevation in leads II, III and a VF. ECPR was started after traditional cardiopulmonary resuscitation, and coronary angiography was performed with the support of extracorporeal membrane oxygenation (ECMO). The left and right coronary arteries were slender and narrow, which was relieved after the injection of 100 µg nitroglycerine through the left coronary artery. After performing a coronary angiogram, the patient was given long-acting nitrates and calcium channel blockers orally, and her chest pain did not reoccur. The patient was weaned from ECMO support after 4 days.

**Conclusion:**

This clinical case highlights the challenges that clinicians face in accurately diagnosing and possibly treating AUB and coronary vasospasm-induced acute myocardial infarction because of its rare occurrence and serious adverse events. ECPR can effectively improve the success rate of cardiopulmonary resuscitation.

## Introduction

Cardiac arrest during pregnancy is receiving increasing attention (1). The most common causes of cardiac arrest associated with pregnancy or delivery are described by the American Heart Association as BEAU-CHOPS (2): bleeding, embolism, aesthetic complications, uterine atony, cardiovascular diseases, hypertension, others, placenta and sepsis. Abnormal uterine bleeding (AUB) is a common symptom and disease in obstetrics and gynecology and increases maternal morbidity and mortality for pregnant women with preexisting AUB-related anemia (3, 4). However, there are few reports on cardiac arrest in nonpregnant women caused by AUB (5).

Extracorporeal CPR (ECPR) refers to the initiation of cardiopulmonary bypass during the resuscitation of a patient in cardiac arrest. The 2023 American Heart Association Focused Update.

The Adult Advanced Cardiovascular Life Support recommends the use of ECPR for patients with cardiac arrest refractory to standard ACLS (6). In this case, we report ECPR used in a nonpregnant woman with cardiac arrest caused by coronary vasospasm and AUB.

### Patient presentation

**Figure 1:**
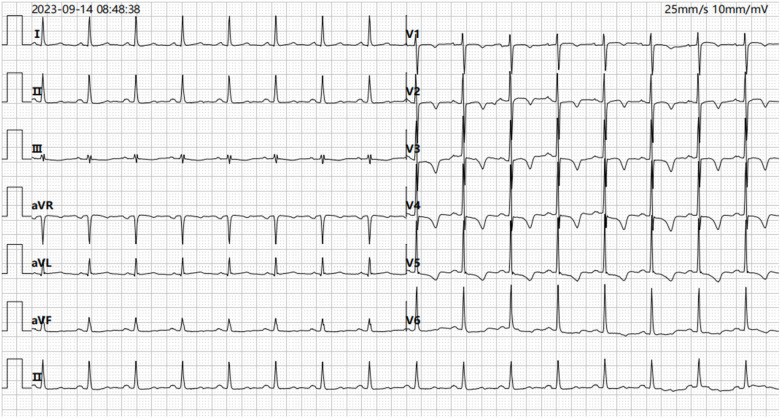
ECG recorded on admission demonstrating T wave changes in leads V2, V3, V4, and V5 (ECG, electrocardiogram).

**Figure 2:**
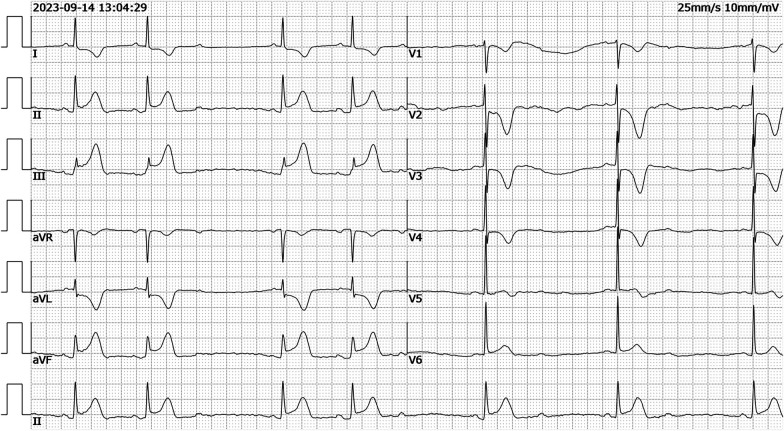
ECG recorded demonstrating ST-segment elevation in leads II, III, aVF which limb leads is type 1 s-degree atrioventricular block (Wenckebach) and chest leads is 2:1 s-degree atrioventricular block (ECG, electrocardiogram).

**Figure 3:**
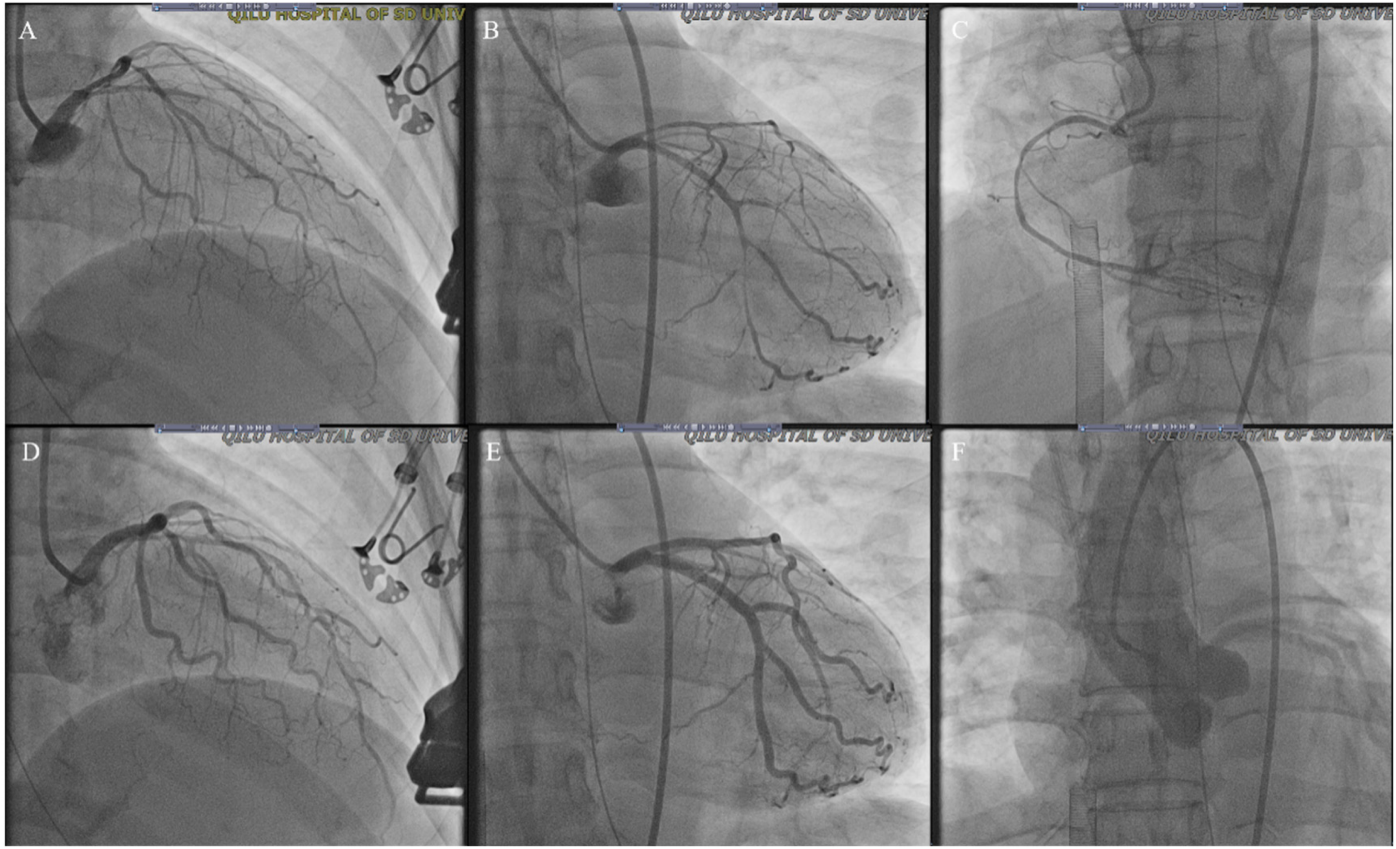
Coronary angiogram from the current stay. **(A–C)** Diffuse slender of LAD, LCX and RCA **(D,E)** improved LAD and LCX spasm after intracoronary injection of 100 ug nitroglycerine; **(F)** showed normal ascending aortography (LAD, left anterior descending artery; LCX, left circumflex artery; RCA, right coronary artery).

**Figure 4:**
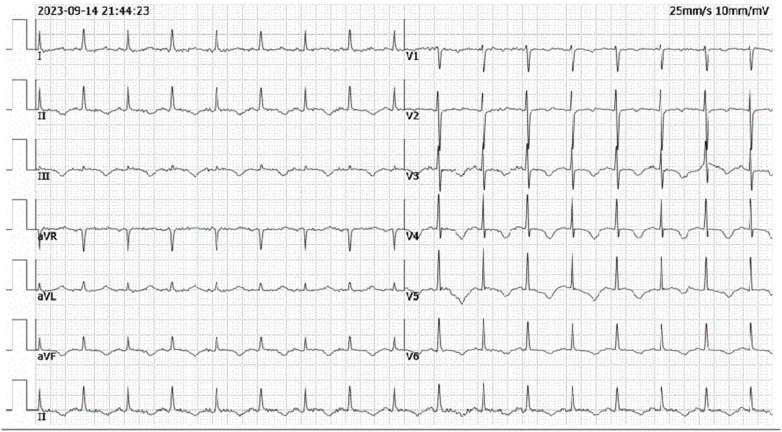
Post-procedural ECG from ICU showing resolution of ST-segment elevation in leads II, III and aVF.

A 52-year-old female was admitted to the emergency room because of sudden chest pain and chest tightness, with a history of hypertension, coronary heart disease and abnormal uterine bleeding for 20 days. She had multiple previous episodes of chest distress and chest pain after activity that coronary computed tomography angiography showed coronary atherosclerosis. The patient had history of taking aspirin, isosorbide mononitrate sustained-release tablets, atorvastatin, isosorbide mononitrate sustained-release tablets, and stopped aspirin for 1 week because of abnormal uterine bleeding. There's no history of smoking, drug abuse or unprescribed medications. The patient went to the gynecology outpatient service 1 day before the disease's onset. Laboratory examination revealed beta-human chorionic gonadotropin (β-HCG), 0 mIU/ml, and hemoglobin, 70 g/L. Ultrasound revealed multiple myomas of the uterus and uneven thickening of the endometrium. Iron polysaccharide complex capsules were given to correct anemia. Echocardiography was performed 2 days prior, and the left ventricular ejection fraction was 72%. The patient repeatedly experienced chest pain and discomfort at 1 a.m., accompanied by chest tightness and sweating. When she entered the emergency room at 9 a.m., her vital signs were as follows: blood pressure of 128/96 mmHg (right arm, supine position), heart rate of 99 bpm, respiratory rate of 15/min, oxygen saturation of 99% and temperature of 36.6°C. Her cardiac troponin level was totally normal. ECG revealed T wave changes in leads V2, V3, V4, and V5 (Figure 1). Vital sign monitoring, nasal catheter oxygen inhalation (3 L/min), and coronary vasodilator (nicorandil 12 mg intravenous drip) were performed. Considering the AUB, antiplatelet drugs were not given at the first time. Cardiology experts recommend a short-term reexamination of ECG and cardiac troponin to assess the indications for early coronary angiography. The patient's symptoms of chest pain were relieved after treatments. The patient's chest pain reappeared at noon and could not be relieved continuously. During the reexamination of the ECG, the patient suddenly experienced loss of consciousness, sweating, and a decreased heart rate. ECG revealed ST-segment elevation in leads II, III and the AVF which limb leads is type 1 s-degree atrioventricular block (Wenckebach) and chest leads is 2:1 s-degree atrioventricular block (Figure 2). The troponin level was 0.016 ng/ml, which was considered acute myocardial infarction. She was given a loading dose of aspirin 300 mg and clopidogrel 300 mg orally according to the cardiology consultation. Bedside ultrasound revealed that left ventricular systolic function decreased diffusely to less than 10%, and the femoral artery was untouched. Endotracheal intubation, isoproterenol, and norepinephrine were given immediately. After that, the patient underwent episodes of cardiac arrest with pulseless electrical activity, and ECPR was started after traditional cardiopulmonary resuscitation. The ICU team placed VA-ECMO beside the bed to restore spontaneous circulation. The vital signs were as follows: blood pressure of 117/95 mmHg (right arm, supine position, 0.4 µg/kg/min norepinephrine), heart rate of 131 bpm, controlled ventilation at 14/min, and a temperature of 36.1°C. The initial setting parameters of ECMO were as follows: rotation speed of 2,780 r, flow rate of 3.63 L/min, gas flow rate of 3 L/min, oxygen concentration of 50%, and water tank temperature of 36°C. The blood gas analysis showed that the patient's hemoglobin had dropped to 49 g/L at 3:40 p.m. and we gave blood transfusion immediately at 4 p.m. The patient underwent urgent coronary angiography. Figure 3 revealed a diffuse slenderness of the coronary artery and severe narrowing of the left circumﬂex (LCX) arteries, in which the heaviest stenosis was 90%. Suspecting spasm, the left coronary artery was injected intracoronary with 100 µg nitroglycerine, which completely relieved the spasm. After relief of spasm, angiography showed left anterior descending artery (LAD) had atherosclerosis with no significant stenosis or occluding lesions, which in good blood flow. The inferior myocardial infarction was confirmed to be caused by coronary vasospasm, and the postoperative electrocardiogram (Figure 4) revealed that the ST segment returned to baseline. The cardiac troponin levels obtained 4 and 12 h later were 2.1 ng/ml and 2.2 ng/ml, respectively.

After the patient entered the ICU, the central venous pressure (CVP) was 9 mmHg. The arterial blood gas test results revealed a pH of 7.32, PaCO2 of 26.1 mmHg, PaO2 of 122 mmHg, and lactic acid level of 8.5 mmol/L. The differential pressure of arterial and central venous carbon dioxide was 3.4 mmHg, and the central venous oxygen saturation was 54%. On the one hand, we strengthened sedation and analgesia and implemented target temperature management to reduce oxygen consumption. On the other hand, the patient received a blood transfusion to increase the oxygen supply. On the second day after the operation, blood gas analysis revealed that the pH, central venous oxygen saturation and lactic acid levels stabilized and improved. During ECMO treatment, the vasoactive agents controlled the mean arterial pressure above 75 mmHg, and the level of lactic acid decreased and returned to normal. The results of the hemodynamic monitoring of the patient during ECMO support are shown in Table 1. The patient was given 100 mg/d aspirin, 20 mg/n atto vastatin, 10 mg/d ezetimibe, 24 mg/h nicorandil, 50 mg/d isosorbide mononitrate, and 90 mg/d diltiazem. As the patient experienced a decreasing trend in her cardiac troponin level, she had no recurrent chest pain, and her left ventricular ejection improved to 55%. With improvements in cardiopulmonary conditions, the number of ECMO settings gradually decreased. ECMO support was removed at 4 days after admission, and weaning from mechanical ventilation was successful after 5 days.

**Table 1:** The hemodynamic monitoring of patient during ECMO support.

Date	9.14 17:16	9.15 01:00	9.15 09:00	9.15 17:00	9.16 09:00	9.17 09:00	9.18 09:00
HR, bpm	131	87	90	99	100	89	97
ABP, mmHg	117/95	91/66	128/72	141/87	127/73	128/86	150/90
NE, ug/kg/min	0.4	0.1	–	–	–	–	–
Dob, ug/kg/min	–	–	0.2	–	–	–	–
CVP, mmHg	9	8	10.9	8	7	12	8
pH	7.32	7.47	7.35	7.3	7.40	7.43	7.39
P(v-a)CO2 Gap, mmHg	3.4	7.7	-	6.4	1.3	9.9	7.6
ScvO2, %	54	74.7	–	68.1	64 5	70.1	63.7
Lac, mmol/L	8.5	2.8	3.2	1.3	0.8	1.0	0.7

HR, heart rate; ABP, arterial blood pressure; NE, norepinephrine; Dob, dobutamine; CVP, central venous pressure; pH, power of hydrogen; P(v-a) CO2 gap, differential pressure of arterial and central venous carbon dioxide; ScvO2, central venous oxygen saturation; Lac, lactic acid.

The patient experienced intermittent vaginal bleeding, and the amount of vaginal bleeding was approximately 50–600 ml per day. The patient was diagnosed with adenomyosis, and the hemoglobin level returned to 105 g/L after treatment. However, there was no tangible effect after intramuscular injection of 10 U oxytocin and infusion of red blood cells, with hemoglobin decreasing to 77 g/L. Uterine artery embolization was performed on the 8th day, and the amount of vaginal bleeding decreased significantly after the operation. Hemoglobin increased to 90 g/L after the operation. After 13 days, with no recurrent chest pain and in stable condition, the patient was transferred from the ICU to the regular cardiology ward and discharged 21 days after the operation.

## Discussion

Here, we describe a case of cardiac arrest due to coronary vasospasm and AUB. She had recurrent chest pain and tightness, but no abnormalities were detected via electrocardiogram, echocardiography, or myocardial injury markers when symptoms did not occur. The patient subsequently suddenly experienced cardiac arrest and was able to regain spontaneous circulation with VA-ECMO support. Coronary angiography confirmed diffuse spasms in multiple coronary arteries. Therefore, we believe that coronary vasospasm and type 2 myocardial infarction caused by anemia were the main causes of cardiac arrest in this patient.

To the best of our knowledge, there are few reports on cardiac arrest in nonpregnant women caused by AUB. Takeshi Yagi reported a 57-year-old woman who experienced cardiac arrest due to massive hemorrhage from uterine adenomyosis with leiomyoma (5). The author suggested that cardiac arrest was caused by hemorrhagic shock.

In patients with known or presumed CAD, acute stressors such as bleeding, tachyarrhythmia, hypoxia or hypotension may result in myocardial injury. According to the fourth universal definition of myocardial infarction, the pathophysiological mechanism leading to ischemic myocardial injury in the case of a mismatch between oxygen supply and demand has been classified as type 2 myocardial infarction (7). The myocardial oxygen supply/demand imbalance attributable to acute myocardial ischemia may be multifactorial and related either to reduced myocardial perfusion due to fixed coronary atherosclerosis without plaque rupture, coronary vasospasm, coronary microvascular dysfunction, coronary embolism, coronary artery dissection with or without intramural hematoma or other mechanisms that reduce the oxygen supply or increase the myocardial oxygen demand (8).

It appears advisable in the acute setting to treat the underlying ischemic imbalance of oxygen supply and demand. This treatment may include volume adjustment, blood pressure management, the administration of blood products, heart rate control, and respiratory support (9, 10). In this case, the patient experienced recurrent symptoms of chest pain that culminated in cardiac arrest, possibly related to anemia that was not corrected in time.

Extracorporeal cardiopulmonary resuscitation (ECPR) is a resuscitation mode that can effectively improve the success rate of cardiopulmonary resuscitation in patients with cardiopulmonary arrest, especially in-hospital cardiopulmonary arrest. In the treatment of cardiac arrest patients, the spontaneous circulation recovery rate is as high as 95% (11), and the discharge survival rate is as high as 27.6%–50% (11–13). The 2023 American Heart Association Focused Update on Adult Advanced Cardiovascular Life Support recommends the use of ECPR for patients with cardiac arrest refractory to standard ACLS (5).

There are currently no data on the incidence and mortality of myocardial infarction induced by AUB. Therefore, type 2 myocardial infarction caused by abnormal uterine bleeding may be underrecognized. In the treatment of cardiac arrest caused by type 2 myocardial infarction, it is very important to correct predisposing factors such as anemia as soon as possible.

## Data Availability

The raw data supporting the conclusions of this article will be made available by the authors, without undue reservation.
